# Magnetic-Based Enrichment of Rare Cells from High Concentrated Blood Samples

**DOI:** 10.3390/cancers12040933

**Published:** 2020-04-10

**Authors:** Junhao Wu, Katharina Raba, Rosa Guglielmi, Bianca Behrens, Guus Van Dalum, Georg Flügen, Andreas Koch, Suraj Patel, Wolfram T. Knoefel, Nikolas H. Stoecklein, Rui P. L. Neves

**Affiliations:** 1 Department of General, Visceral and Pediatric Surgery, University Hospital and Medical Faculty of the Heinrich-Heine University Düsseldorf, Moorenstr. 5, 40225 Düsseldorf, Germany; JunHao.Wu@med.uni-duesseldorf.de (J.W.); Rosa.Guglielmi@med.uni-duesseldorf.de (R.G.); Bianca.Behrens@med.uni-duesseldorf.de (B.B.); vandalum@gmail.com (G.V.D.); georg.fluegen@med.uni-duesseldorf.de (G.F.); WolframTrudo.Knoefel@med.uni-duesseldorf.de (W.T.K.); 2 Institute for Transplantation Diagnostics and Cell Therapeutics, University Hospital and Medical Faculty of the Heinrich-Heine University Düsseldorf, 40225 Düsseldorf, Germany; Katharina.Raba@med.uni-duesseldorf.de; 3 Thermo Fisher Scientific, Postfach 200152, Frankfurter Str. 129B, 64293 Darmstadt, Germany; andreas.koch@thermofisher.com; 4 Thermo Fisher Scientific, 3 Fountain Drive, Inchinnan, Renfrew PA4 9RF, UK; suraj.patel@thermofisher.com

**Keywords:** circulating tumor cells, immunomagnetic enrichment, concentrated blood products, diagnostic leukapheresis, IsoFlux, KingFisher

## Abstract

Here, we tested two magnetic-bead based systems for the enrichment and detection of rare tumor cells in concentrated blood products. For that, the defined numbers of cells from three pancreatic cancer cell lines were spiked in 10^8^ peripheral blood mononuclear cells (PBMNCs) concentrated in 1 mL, mimicking diagnostic leukapheresis (DLA) samples, and samples were processed for circulating tumor cells (CTC) enrichment with the IsoFlux or the KingFisher systems, using different types of magnetic beads from the respective technology providers. Beads were conjugated with different anti-EpCAM and MUC-1 antibodies. Recovered cells were enumerated and documented by fluorescent microscopy. For the IsoFlux system, best performance was obtained with IsoFlux CTC enrichment kit, but these beads compromised the subsequent immunofluorescence staining. For the KingFisher system, best recoveries were obtained using Dynabeads Biotin Binder beads. These beads also allowed one to capture CTCs with different antibodies and the subsequent immunofluorescence staining. KingFisher instrument allowed a single and streamlined protocol for the enrichment and staining of CTCs that further prevented cell loss at the enrichment/staining interface. Both IsoFlux and KingFisher systems allowed the enrichment of cell line cells from the mimicked-DLA samples. However, in this particular experimental setting, the recovery rates obtained with the KingFisher system were globally higher, the system was more cost-effective, and it allowed higher throughput.

## 1. Introduction

A growing body of evidence suggests that circulating tumor cells (CTCs), i.e., tumor cells shed into the circulation by solid tissues, have the potential to be used as biomarkers for clinical monitoring and as a source of information to better understand the complex metastatic cascade [1,2]. However, assessing the full clinical and biological informative value of CTCs has remained very challenging due to their rarity [3,4]. Considering that typically only 1–10 CTCs are present in 1 mL of blood [4], the low blood volume of standard blood samples (7.5–10 mL) strongly contributes to the low detection frequency [5]. Diagnostic leukapheresis (DLA) is a powerful approach to sample liters of blood [6] improving significantly the frequency and numbers of detected CTCs [6,7,8,9]. DLA is based on a continuous flow centrifugation of peripheral blood leading to a density-based separation of the cellular fractions which can be selectively harvested. The principle underlying application of DLA to enrich CTCs is that CTCs have a similar density and can be harvested together with the faction of peripheral blood mononuclear cells (PBMNCs) from patients [4]. As a result, DLA products typically contain a concentration of PBMNCs that is >25× higher than the one found in peripheral blood. This is currently the major challenge to effectively utilize the full power of DLA for CTC detection and isolation [4,7].

Most commonly, CTC-detection relies on the immunomagnetic enrichment of cells expressing epithelial cell adhesion molecule (EpCAM) [10], followed by the immunofluorescence detection of cytokeratin (CK) positive and CD45 negative nucleated (CK^pos^/CD45^neg^/DAPI^pos^) cells [3,11]. This is the basis of the CellSearch system [12,13], the only FDA-cleared system for CTC enrichment and which we and others have previously demonstrated to be efficient to process DLA samples [6,7,8,9]. However, the throughput for DLA is limited [4] and the enrichment of CTCs from epithelial malignancies is restricted to EpCAM [14]. One alternative technology for positive CTC enrichment is the IsoFlux system (Fluxion Biosciences Inc., Alameda, CA, USA) [15], to our best knowledge the only semi-automated bead-based immunomagnetic system commercially available. IsoFlux was demonstrated to be effective for EpCAM-based enrichment of breast, prostate [15], and colorectal [16] cell line cells, and CTCs from patients with prostate [15,17], hepatocellular carcinoma [18], and bladder cancer [19]. This microfluidic platform offers some flexibility, since magnetic streptavidin-conjugated beads are available to broaden the spectrum of epitopes that can be used for enrichment. However, the staining of enriched cells for CTC-detection is performed manually, which can be disadvantageous for the reproducibility and throughput of the system. In the present work, we compared the performance of this system to enrich tumor cells from samples mimicking DLA products with the one from the KingFisher Duo Prime Purification System (Thermo Fisher Scientific Inc., Waltham, MA, USA), an instrument present in the market for magnetic-based nucleic acid purification [20] immunoprecipitation and protein purification [21], but which was never reported for isolation of rare cells. The instrument uses permanent earth magnetic rods to transfer magnetic bead-bound samples through successive solutions according to user-defined programs, and its specifications suggested to us that the system could also be used for enrichment of CTCs. Moreover, the specifications also suggested that different epitopes and magnetic beads could be used, and that enrichment and staining steps could be combined in one single automatic protocol, possibilities that, combined, are not available in any commercial system for CTC enumeration.

The use of different epitopes for CTC enrichment is of particular interest in the case of tumors as pancreatic ductal adenocarcinomas (PDAC), in which the level of EpCAM expression is particularly heterogeneous [22] and low in approximately 50% of the cases [23,24]. This may explain the relative low number [22,25,26] and frequency [25] of CTCs detected with EpCAM-based assays, particularly when compared to other metastatic diseases [13] and to EpCAM-independent assays [22,27,28]. Aiming at strategies to overcome the limitation of using EpCAM as single epitope for enrichment of CTCs, we tested both IsoFlux and KingFisher systems using different EpCAM- and Mucin1 (MUC-1)-coupled magnetic beads to enrich pancreatic tumor cells. MUC-1 is a transmembrane glycoprotein, which is highly expressed in the majority of pancreatic tumors [29], and that was already proposed as a therapeutic target [30,31]. A previous work has shown that high numbers of MUC-1^pos^/EpCAM^pos^ CTCs correlate with shorter overall survival in patients with pancreatic cancer [32], and data suggest that MUC-1 and EpCAM might identify different subtypes of CTCs in pancreatic [33], but also in ovarian [34,35] and metastatic breast cancer [36].

## 2. Results

### 2.1. Epitope Expression in Model Cells Lines

**Figure 1:**
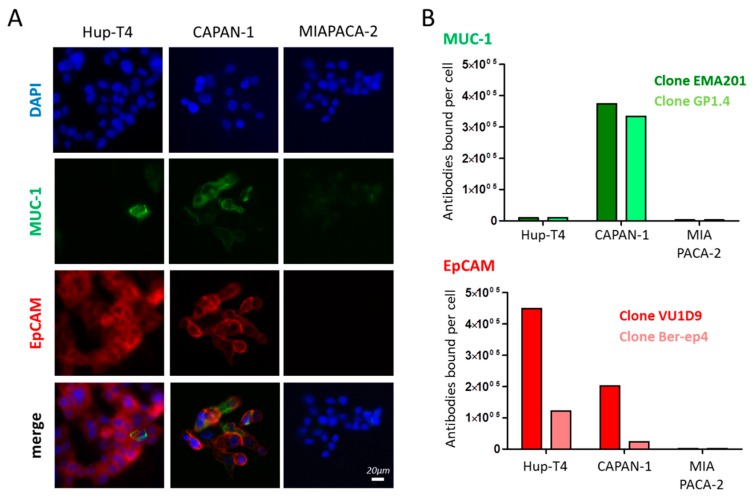
MUC-1 and EpCAM expression in HuP-T4, CAPAN-1 and MIAPACA-2 pancreatic cells lines. (**A**) Immune-fluorescence microscopy analysis of EpCAM and MUC-1 expression. (**B**) Number of epitopes detected by two anti-MUC-1 clones (EMA201 and GP1.4), and two anti-EpCAM clones (VU1D9 and Ber-EP4) by flow cytometry.

To analyze the suitability of model cells for enrichment experiments, we investigated three different human pancreatic cancer cell lines for their EpCAM and MUC-1 surface expression (Figure 1). Based on the levels of the epitopes detected, we have classified the pancreatic line HuP-T4 line as EpCAM^High^/MUC-1^Low^, the CAPAN-1 line as EpCAM^Mid^/MUC-1^High^, and the MIAPACA-2 line as EpCAM^Low^/MUC-1^Neg^. Strikingly, the number of epitopes that we could detect with the Anti-EpCAM VU1D9 clone was higher than with the clone BerEP4. Clear MUC-1 expression could only be detected on CAPAN-1 cells and the number of epitopes detected by the two clones tested did not differ considerably (Figure 1).

### 2.2. Beads Used for Enrichment and Read-Out for Cell Enumeration

For enrichment in the Isoflux system, we used three different types of beads available from Fluxion (Iso-CEK, IsoRCEK, and Iso-RCEK-SA) and according to the instructions provided by the manufacturer (Table 1). As no protocols or standards were available for enrichment in the KingFisher system, we tested four different types of beads available from Thermo Fisher Scientific (Dy-EpE, Dy-ACK, Dy-BioB, and Pi-Strep) and tested three different amounts of those beads (minimal (MIN), middle (MID) and maximal (MAX)) (Table 1).

**Figure 2:**
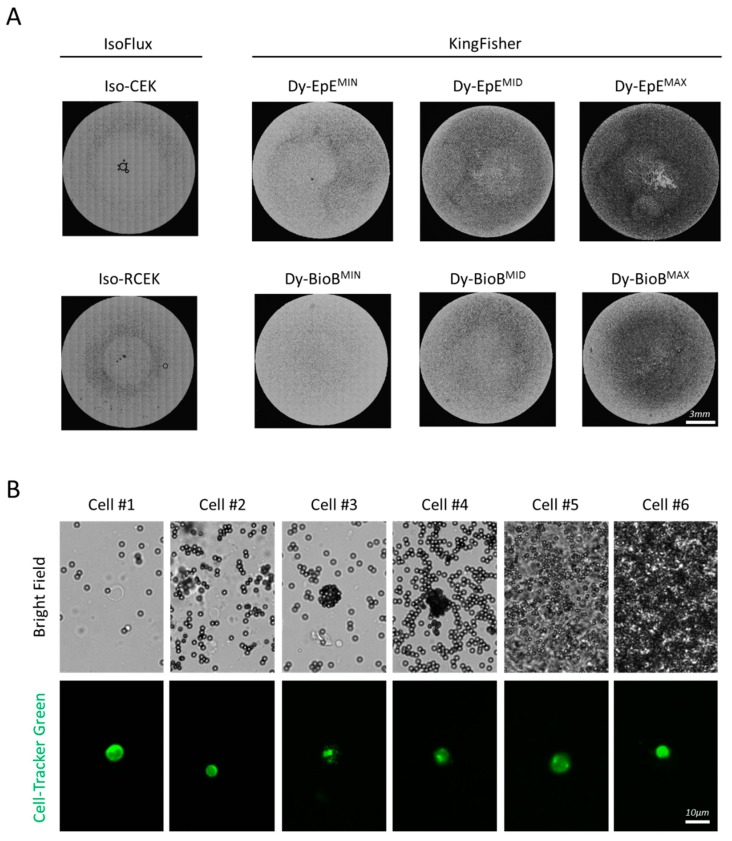
Identification of enriched pre-labelled cells among the beads. (**A**) Distribution of Iso-CEK, Iso-RCEK, Dy-EpE^MIN^,^MID^,^MAX^ and Dy-BioB^MIN^,^MID^,^MAX^ beads in field of a three-field microscope slide used for enumeration of enriched cells. Each image is a montage of all 357 tiled bright field images covering the complete field. (**B**) Six individual cells identified in one same sample enriched with Dy-EpE beads.

We defined the MIN amount as the number of Thermo Fisher beads, providing the same surface area as the Iso-CEK beads in the standard IsoFlux CTC Enrichment Kit assay. Using flow cytometry, we determined the size of the Iso-CEK beads as 4.2 µm (See Figure S1) and analyzing their spectrophotometric characteristics, we estimated that 10.98 × 10^5^ beads are present in the 40 µL of bead suspension used per sample in the IsoFlux CTC Enrichment Kit assay (See Figure S2). Based on these values, we calculated that the surface provided by these beads in the respective assay is 6.11 × 10^7^ µm^2^ (see Figure S2D) and determined the volume of Thermo Fisher beads necessary to achieve that surface based on their sizes and concentrations given by the manufacturer. For each type of Thermo Fisher beads, we subsequently defined the middle (MID) amount of beads as 5× MIN, and the maximal (MAX) amount of beads as 10× MIN (see Table S1). These different amounts of beads were clearly distinct under the bright-field microscope light (Figure 2A), however they did not compromise the identification of fluorescent-labeled cells, even in areas of the slide-field where the concentration of beads was highest (typically the center of the sample field) (Figure 2B).

### 2.3. EpCAM-Based Enrichment of Spiked Cells

**Figure 3:**
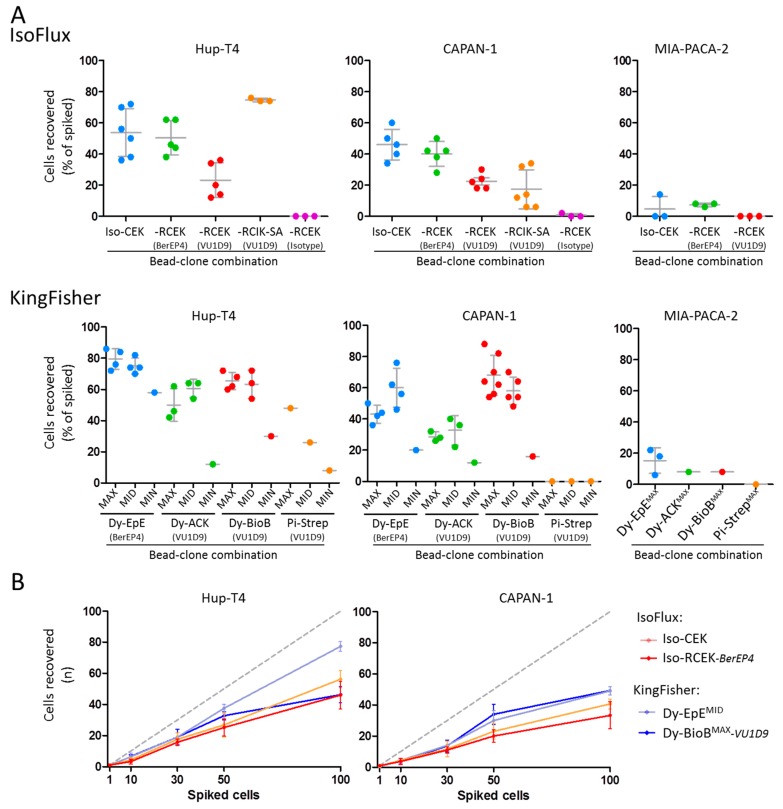
EpCAM-based recovery of HuP-T4, CAPAN-1 and MIA-PACA-2 cells spiked in mimicked-DLA products. (**A**) Recovery of 50 pre-labeled cells from the three lines using the IsoFlux system and three types of beads available from Fluxion (Iso-CEK, Iso-RCEK, and Iso-RCIK-SA) (upper panels) and using the KingFisher Duo system running the WuDuo1 program three different amounts (“MAX”, “MID” and “MIN”) of four types of beads available from Thermo Scientific (Dy-EpE, Dy-ACK, Dy-BioB, and Pi-Strep) (lower panels). (**B**) Recovery of different numbers of spiked pre-labeled HuP-T4 and CAPAN-1 cells using Iso-CEK and Iso-RECK-*BerEP4* beads in the IsoFlux system, and Dy-EpE^MID^ and Dy-BioB^MAX^-*VU1D9* beads in the KingFisher Duo system.

After proving the suitability of the mimicked-DLA products to model patient-derived DLAs [37] and defining different amounts of beads for enrichment, we challenged the IsoFlux and KingFisher systems for EpCAM-based enrichment of pancreatic cells pre-labeled with CellTracker Green spiked in mimicked-DLA products (Figure 3). The IsoFlux system was used with its standard enrichment program, while for the KingFisher system we designed a first protocol with one enrichment and one washing step, the WuDuo1 program (See Figure S3).

Using both systems, we could recover cells from the three pancreatic lines, and for each bead type used the recoveries were globally concordant with the level of EpCAM expression in the cells: HuP-T4 cells were most efficiently recovered, followed by CAPAN-1 and lastly by MIAPACA-2 (Figure 3A). The highest mean recoveries of HuP-T4 and CAPAN-1 cells were obtained in the KingFisher system with Dy-EpE beads and Dy-BioB beads, respectively (Figure 3A,B). In both cases, these mean recoveries were in line or even higher than the ones that we obtained with the CellSearch system (See Figure S4). No statistically significant differences could be detected between recovery rates obtained using the MID and MAX amounts of beads (Figure 3). In the IsoFlux system, Iso-CEK and Iso-RCEK-*BerEP4* beads were the ones with more consistent results. Interestingly, the recoveries with Iso-RCEK-*BerEP4* beads were consistently higher than recoveries with the Iso-RCEK-*VU1D9,* despite the higher abundance of the VU1D9 epitope on the cells (Figure 1).

Based on these results, we further tested the Iso-CEK, Iso-RCEK-*BerEP4*, Dy-EpE^MID^, and Dy-BioB^MAX^-*VU1D9* beads, to recover different amounts of HuP-T4 and CAPAN-1 cells spiked in mimicked-DLA samples (1–100 cells) (Figure 3B). Additionally, in this set of experiments, the recovery of HuP-T4 cells (43%–78%) was globally more efficient than CAPAN-1 cells (34%–52%) (see Figure S5), and with the exception of one measurement with Dy-BioB^MAX^-*VU1D9* (100 cells), higher recoveries were obtained using the Dynabeads in the KingFisher system. Importantly, in the range tested, the recoveries for both CAPAN-1 and HuP-T4 lines in both systems were close to linearity (R^2^ of linear regression were between 0.8411 and 0.9913) (see Figure S5).

Notably, the EpCAM-based enrichment of CAPAN-1 cells was differentially influenced by cell preservatives. CellSave and TransFix fixatives positively influence the recovery in both systems, PFA 0.1% significantly decreased the recovery in both systems, and Streck tubes caused a striking reduction in recovery with Iso-CEK beads, but not with the Dy-BioB^MAX^-*VU1D9* beads (see Figure S6). The positive effect of TransFix preservative could also be recapitulated in experiments using CAPAN-1 cells spiked in normal whole blood samples (see Figure S7A).

Using the Dy-BioB^MAX^-*VU1D9* beads in the KingFisher system, we could also recover HCT-116, SW620 (both colorectal cancer) and SKBR-3 (breast cancer) cells, showing that the system can also be applied for other tumor entities (See Figure S7B). In additional experiments, in which we used Hoechst nuclear dye to also detect the WBCs co-enriched using the Dy-BioB^MAX^-*VU1D9* beads and the WuDuo1 program in the KingFisher system, we detected, on average, 18061 WBCs. This indicates a depletion efficiency of 3.7 Logs, corresponding to a depletion of >99.98% of WBCs and it results in an estimated CTC purity of 0.188% (See Figure S8).

### 2.4. Alternative Strategies for Enrichment of CTCs with the KingFisher System

**Figure 4:**
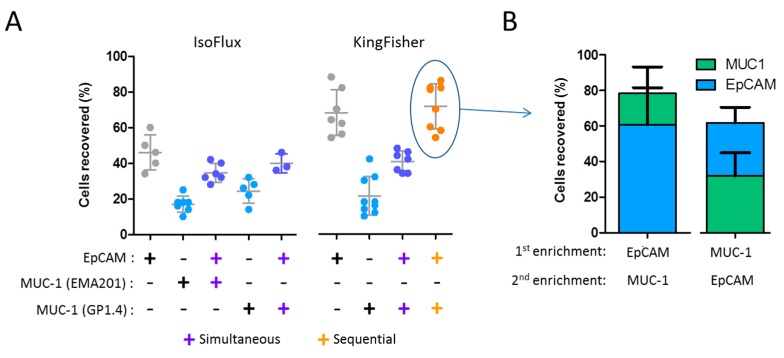
MUC-1 alone and MUC-1/EpCAM combined recovery of CAPAN-1 cells spiked in mimicked-DLA products. (**A**) (Left panel) Recovery of 50 pre-labeled CAPAN-1 cells with Iso-RCEK beads coupled with anti-MUC-1 clones EMA201 and GP1.4 alone or in combination (simultaneous) with anti-EpCAM coupled beads using the IsoFlux system. For the simultaneous MUC-1 and EpCAM recovery, half of the amount of each bead type was used, so that the total amount of beads in the experiment was according to the original protocol. Data in grey are the same as in Figure 2. (Right panel) Recovery of 50 pre-labeled CAPAN-1 cells with Dy-BioB beads coupled with the GP1.4 clone alone or in combination (simultaneously and sequentially) with Dy-BioB anti-EpCAM coupled beads using the KingFisher Duo system. For the simultaneous MUC-1 and EpCAM recovery, half of the amount of each bead type was used, so that the total amount of beads in the experiment was the same as described in the material and methods (Dy-BioB^MAX^). Data in grey are the same as in Figure 2. (**B**) Recovery of 50 pre-labeled CAPAN-1 cells after sequential EpCAM- and MUC-1-based enrichment in the KingFisher system.

We tested MUC-1 as an alternative or additional marker for the enrichment of pancreatic cells using Dy-BioB^MAX^ and Iso-RCEK beads in their respective systems (Figure 4).

Interestingly, exclusively MUC-1-based recovery rates were consistently and significantly lower compared to those previously obtained with the same beads coupled with the VU1D9 or BerEP4 anti-EpCAM clones (Figure 4A). This is more surprising given the higher number of MUC-1 epitopes compared to EpCAM in CAPAN-1 cells (Figure 1). Combining MUC-1- and EpCAM-coupled beads in the same enrichment step (simultaneous enrichment), we could only partially increase the recovery rate. Yet, more interestingly, using the WuDuo2 protocol, it was possible to perform sequential MUC-1- and EpCAM-based enrichments in the KingFisher, and thus achieve global recovery rates similar to the ones obtained with EpCAM alone, while capturing two separate populations of cells (Figure 4B). Taking advantage of the flexibility of the KingFisher system, we have also tested the recovery rate of CAPAN-1 cells performing CD45 depletion, followed by EpCAM-based enrichment in a single automated protocol (See Figure S9). Despite achieving a reduction of the number of background mono nuclear cells of almost 80% with the initial CD45-based depletion, we could not improve the subsequent recovery rate of CAPAN-1 cells.

### 2.5. Staining of Enriched Cells

**Figure 5:**
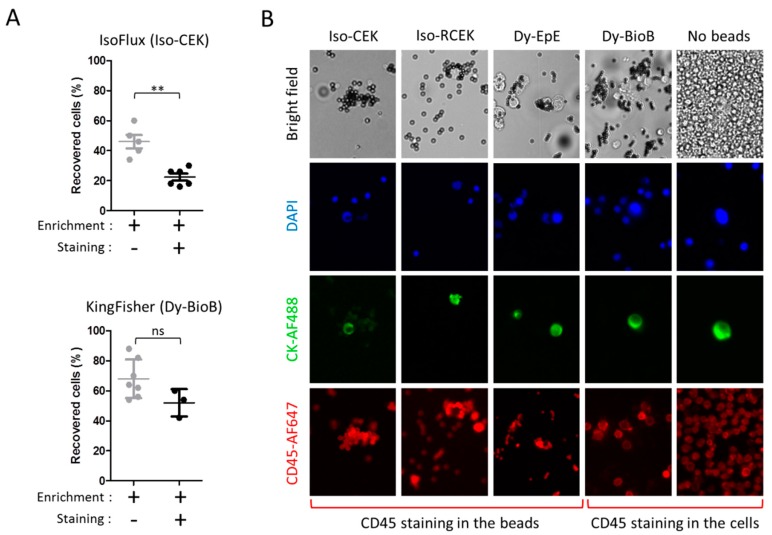
Impact of staining in the recovery and identification of cells spiked in mimicked-DLA products. (**A**) Impact of the staining procedure. *(Upper panel)* Recovery of 50 pre-labeled CAPAN-1 cells with Iso-CEK beads, with and without the subsequent staining procedure performed according to the IsoFlux protocol. For this experiment, the fluorescent-conjugated antibodies in the respective protocols were replaced by non-conjugated mouse IgG isotype control. *(Lower panel)* Recovery of 50 pre-labeled CAPAN-1 cells with Dy-BioB^MAX^ beads, with and without a subsequent staining procedure performed automatically in the KingFisher (protocol *WuDuo Staining*). (**B**) Impact of beads in the immunofluorescence identification of spiked and hematogenous cells. Cells enriched with the Iso-CEK, Iso-RCEK, Dy-EpE, and Dy-BioB beads were stained with DAPI, and AF488-conjugated CKs, and AF647-conjugated CD45 mouse monoclonal antibodies.

Next, we tested the impact of the staining procedure necessary for CTC enumeration in clinical samples after enrichment (Figure 5).

Simulating the staining according to the IsoFlux Circulating Tumor Cell Enumeration Kit protocol, but using cells pre-labeled with CellTracker Green, we observed an additional reduction of 52% in the mean recovery of CAPAN-1 cells (from 46% to 22%, p = 0.0078 Mann–Whitney test), while a staining step introduced in the KingFisher WuDuo1 protocol (WuDuo1S protocol) led to a much milder reduction of 24% (from 68% to 52%, p = 0.0864 Mann–Whitney test) (Figure 5A). Interestingly, the introduction of this staining step, in which the cells are passed by one more solution than in the previous WuDou1 protocol, substantially reduced the number of WBCs co-enriched to a mean of 7588. This increased the depletion efficiency to 4.1 Logs, corresponding to a depletion of >99.99% of WBCs and consequently had a positive impact in the estimated purity (0.346%) (see Figure S8).

Notably, when antibody-based staining was effectively performed, we observed an increase in the fluorescence intensity of the Iso-CEK, Iso-RCEK-*VU1D9*, and Dy-EpE beads itself, particularly in the AF647-CD45 channel, suggesting that these beads retain capacity to unspecifically capture the staining antibodies (Figure 5B). Although the identification of CK^pos^ events was still possible, capturing of the staining antibodies by the beads created major difficulties to reliably exclude the presence of CD45 staining from those events and even to identify hematogenous cells expected to be CD45^pos^. Capturing of the antibodies by these beads could be further validated by flow cytometry (see Figure S10). The exception were Dy-BioB beads, for which no binding of staining antibodies was observed by microscopy and flow cytometry, as expected, due to the fact that coupling to the Dy-BioB beads is dependent on biotin and this is not present in any of the staining antibodies.

## 3. Discussion

Although DLA allows sampling liters of blood from tumor patients significantly augmenting CTC yield [6,7,8], the excess of WBCs in DLA products is challenging for effective CTC detection and currently limits the volume of DLA product that can be used. Previously, we have demonstrated upon analysis of only 2 mL with the CellSearch system, that DLA products contain higher concentrations and numbers of CTCs than those found in standard blood samples [6,8], and in a multicenter European study, we have started to uncover the potential of analyzing larger volumes of product [7]. An analysis of the complete DLA product (typically >40 mL) could provide an unprecedented opportunity to obtain enough CTCs for a more systematic molecular and functional characterization of the systemic disease towards a real liquid biopsy [7]. The fractioning of DLA products for the parallel processing of multiple aliquots is not practically or economically viable, considering the actual costs per assay of the so far described technologies. Therefore, workflows allowing the cost effective and higher throughput processing of highly concentrated DLA products are of great need. In this context, we have evaluated the technical performance of IsoFlux and KingFisher systems to process samples mimicking DLA products containing spiked pancreatic cell line cells. These two systems are, to our best knowledge, the only systems available to perform the semi-automated magnetic-based positive enrichment of CTCs.

Globally, our work indicates that, although the enrichment of rare cells was possible with both systems, the efficiency of the KingFisher system is superior. This is notable, considering the fact that the KingFisher system was originally not designed for this purpose. The two systems differ considerably in their concept, which might explain the differences in performance. In the IsoFlux system, the sample experiences the magnetic field when flowing in a microfluidic channel. As the sample passes only once by the magnet, cells only have one (very short) opportunity to be collected. Differently, in the KingFisher, the sample is kept in a reservoir and the magnetic field is applied by a permanent earth magnetic rod that moves vertically through the sample in a defined number of times. In the basis of all protocols that we designed for the KingFisher, collection was done in three steps of 2′30″ each (i.e., totally the sample is exposed to the magnet for 7.5 min in each collection step). This longer time will favor the capturing procedure. Although in the present work, we demonstrate the feasibility in 1mL samples, the low costs per sample of the KingFisher System (<20 EUR for one-bead type-based enrichment and <8 EUR for staining, according to current list prices), the possibility to run up to 12 aliquots in parallel under the same experimental conditions (scalable to 96 with the KingFisher Flex system), and the inclusion of automatic staining might open new perspectives for processing larger volumes of clinical DLA products.

One other unique feature of both IsoFlux and KingFisher systems is that they are flexible concerning the type of beads and the enrichment epitope. In a first step, we used EpCAM-based enrichment to compare the standard anti-EpCAM pre-coupled beads with self-coupled beads. Our results indicate that the recovery rates obtained with self-coupled beads can be similar to those of the pre-coupled counterparts, despite striking differences in the performance of the different self-coupled beads tested. In both systems, smaller beads (i.e., 3 µm Iso-RCIK-SA and 1 µm Pi-Strep beads) were generally less efficient at capturing spiked cells, indicating the limitations of smaller magnetic particles for cell enrichment under the magnetic momentums of their respective magnets. Although the number of beads that can bind to one same cell is higher if the beads are smaller, the magnetic force exerted in each cell/beads complex is more strongly influenced by the diameter of the beads bound, which explains why larger beads, to a certain extent, allow higher recoveries of cells [38].

Using the KingFisher system we could titrate the amount of beads per assay. The best results were obtained using 248 × 10^5^ Dy-BioB beads (Dy-BioB^MAX^), a number that is ~23 times higher that the number of beads used in the Isofux (See Table S1, Figure S3), and that provide a surface for contact that is 10× larger than that of the Isofux standard assay. This larger surface will favor the binding of beads to the cells. Typically, the conjugation of biotin groups to antibodies is done randomly, resulting in an unequal number and distribution of biotin groups over the antibody molecules. As coupling of the antibodies to beads happens via the biotin groups, the antibodies can be coupled to the DyBioB beads in orientations that hinder ligand binding. Moreover, biotinylation can even diminish the binding capacity of the antibodies, if its Fab regions become indeed biotinylated during this random process [39,40]. As an alternative to this random process, the use of site-specific antibody biotinylation of the Fc domain can maximize the accessibility of the Fab regions of antibodies coupled to surfaces [40]. This was previously demonstrated to increase the capturing of CTCs to a microfluidic chip [39], and to magnetic nanoparticles coated on a micro-sized immune-graphene oxide sheet [38], and we anticipate that the same could further improve CTC capturing capacities of DyBioB beads in the KingFisher system.

In general, recovery was in line with the level of EpCAM-epitope expression in the cells. Interestingly, using Iso-RCEK beads, we could obtain higher recoveries with the Ber-EP4 EpCAM antibody clone than with the VU1D9, despite the higher abundance of VU1D9 epitopes detected in the cell lines, which might indicate significant differences in the affinity of these two antibody clones [41]. Such differences in affinity have an impact on the strength of the bond between cells and beads [42], critical for pulling the cells towards the magnet. Our subsequent MUC-1-based enrichments further reinforce that idea. In PDAC, MUC-1 is an important cellular epitope, and in CAPAN-1 cells, it is much more abundant than EpCAM. Despite that, in both systems, recovery with MUC-1 was dramatically lower compared to that of beads coupled with EpCAM. However, despite the low recovery, the possibility of capturing MUC-1 expressing CTCs seems of particular interest in samples of patients with tumors, as PDAC as assays based in EpCAM alone have resulted in the low number and frequency of CTCs [22,25,26]. Our results indicate that the sequential use of EpCAM and MUC-1 in the KingFisher might increase CTC yield and allow capturing different subpopulations of CTCs, which will deserve further investigation.

Interestingly, fixatives had an impact on the recovery of the spiked tumor cells. The challenge in fixation of samples resides in finding the right compromise between preserving the antigens while maintaining their ability to be reached by antibodies [43]. Paraformaldehyde (PFA) is widely used for immunostaining (IS) (e.g., for fluorescence microscopy) (typically at 4%), but it has been demonstrated to cause loss of epitopes, to sterically hinder the access of the antibodies to their antigens, and the mislocalization of target proteins [44]. In addition, the more distal part of EpCAM molecule, recognized by VU1D9 and BerEP4 antibodies, has been shown to be degraded by protocols for imune histochemistry (IHC) of tissue sections involving formaldehyde fixation (typically at 4%, i.e., 10% Formalin solution) [45]. Although the concentration of PFA used in the present work (0.1%) is far below of that used for IS and IHC, partial degradation and masking of the EpCAM epitope by PFA can explain the drastic negative effect of this fixative on recovery of rare cells. The fixative contained on CellSave, TransFix and Streck tubes are proprietary and therefore their impact on cell recovery is difficult to interpret. However, the information available (e.g., from patent applications) suggests the use of formaldehyde releasers, which keep a concentration of formaldehyde high enough to stabilize cell morphology, but also low enough to mitigate the negative effect on the EpCAM epitope. The distinct effect of Streck fixative on the recovery with Iso-CEK and Dy-BioB-VU1D9 beads might result from the different impact of this fixative on the epitopes recognized by the antibodies coupled to these beads (information not available for the case of Iso-CEK beads). Collectively, our results indicate that the immunoganetic recovery of rare cells in highly concentrated blood samples is determined by an assay specific combination of multiple factors, such as cell size, bead size, epitope expression, sample fixation, antibody affinity and magnetic field, which was more favorable in the case of the KingFisher system.

Similarly, to any other CTC enrichment technology, IsoFlux and KingFisher systems were not able to provide pure CTCs and many hematogenous cells were co-enriched. We have tested immunofluorescence staining to discriminate spiked tumor cells from the background cells, an essential step for CTC enumeration in clinical samples. In the IsoFlux system, this step is performed manually outside the platform. This extended the hands-on time and led to an additional 50% cell loss, which may limit the reproducibility of the technique and its applicability to larger studies. In the KingFisher system, the staining can be automated and integrated with enrichment in one workflow. This resulted in a more effective cell recovery and faster sample processing. Importantly, we have noticed that self-coupled Iso-RCEK beads, pre-coupled Iso-CEK and Dy-EpE beads extensively captured the antibodies used for staining, sequestering them from binding to the cells. The antibodies used here for immunofluorescence staining are mouse monoclonal antibodies (anti-panCK C11, anti-CK19 A53-B/A2, and anti-CD45 HI30) and were chosen due to their extensive clinical validation. These clones are the ones used in the CellSearch system [14], considered the gold standard and still the only system cleared by the Food and Drug Administration (FDA) for the in vitro diagnostic (IVD) enumeration of CTCs in patients. Further tests indicated that the binding of antibodies to Iso-CEK beads was dependent on the *Fc* fraction of mouse immunoglobulin G (IgG) (data not shown). Strategies to block antibody capturing by beads using different protein solutions could reduce, but not completely eliminate, the problem (data not shown), a fact that, from our perspective, limits the use of these beads and particularly the IsoFlux system for CTC enumeration purposes. Interestingly, as alternative to enumeration, immune-magnetically enriched CTCs can be detected with sensitive DNA- or RNA-based assays assays [46,47,48]. Similar assays were already successfully applied in cellular fractions enriched with IsoFlux [15,19,49] and with Dynabeads processed manually [50]. Such strategies overcome the difficulties faced for microscopic enumeration, although they do not allow the generating of individualized molecular profiles of the different CTCs.

## 4. Materials and Methods

### 4.1. Cell Lines, Cell Culture, and Preparation of Spiked Samples

Three pancreatic cancer cells, CAPAN-1, MIAPACA-2 and HuP-T4 were obtained from the Leibniz Institute DSMZ—German Collection of Microorganisms and Cell Cultures (Germany). All cell lines were maintained in culture under standard conditions: CAPAN-1 were cultured in RPMI1640 (PAN-biotech, Aidenbach, Germany) supplemented with 20% fetal bovine serum (FBS) (Sigma, Steinheim, Germany); MIAPACA-2 in Dulbecco’s MEM (PAN-biotech, Germany) supplemented with 20% FBS and 2.5% horse serum (PAN-biotech, Germany); and HuP-T4 in MEM Eagle (with EBSS, 2 mM L-Glutamine, 1 mM Sodium pyruvate, NEAA, and 1.5 g/L NaHCO3) (PAN-biotech, Germany), supplemented with 20% FBS. To prepare single-cell suspensions for experiments, cells were harvested from culture flasks using standard treatment with 0.05% Trypsin (PAN-biotech, Germany). For optimization of the CTC enrichment procedure, we used cells pre-labeled with CellTracker Green CMFDA Dye (Life Technologies, Carlsbad, CA, USA), according to the manufacturer’s protocol. Subsequently, one, ten, or 30 dye positive cells were spiked manually, while 50 or 100 dye positive cells were spiked by flow cytometry using the MoFlo XDP flow cytometer (Beckman Coulter, Germany) into samples mimicking patient DLA products (mimicked-DLA products) (1 mL each sample). These mimicked-DLA products were prepared by isolating PBMNCs from the Buffy coats of healthy donors using Ficoll-Paque PLUS (d = 1.077 ± 0.001 g/mL; GE Healthcare, Sweden) density gradient centrifugation at 800× *g* for 20 min, subsequently washing the cells twice with PBS, and resuspending the cells to a concentration of 10^8^ PBMNCs/mL, with PBS containing 0.5% BSA and 2 mM EDTA. Importantly, the cellular composition of these products was comparable with that of the patient-derived DLA products (see non-published material). All experiments were performed with the approval of the Local Ethics Committee of Medical Faculty of the Heinrich-Heine-University Düsseldorf, Germany (N. 4446). The experiments were performed in accordance with the relevant guidelines and regulations and ethical principles of the Declaration of Helsinki. Buffy coats were obtained from healthy blood donors, as anonymously provided by the blood donation center of the Institute for Transplantation Diagnostics and Cell Therapeutics, University Hospital Düsseldorf, Düsseldorf, Germany, with written informed consent for the use of surplus blood products for research purposes obtained from each blood donor. Data related to human samples were all analyzed anonymously.

### 4.2. Evaluation of MUC-1 and EpCAM Expression on Cell Lines

For the immune-fluorescence microscopy analysis of EpCAM and MUC-1 expression, cells were grown in an 8 well glass Lab-Tek Chamber Slide (Nunc, Rochester, NY, USA). For immune-staining, cells were washed once with PBS, incubated for 45 min with 200 µL of staining mix (AF488-conjugated anti-MUC-1 clone GP1.4 at 3.5 µg/mL (Novus, CO, USA), AF647-conjugated anti-EpCAM clone VU1D9 at 3.5 µg/mL (Cell Signaling Technology, Danvers, MA, USA), in PBS with 10% of AB-Serum (Bio-Rad Medical Diagnostics, Dreieich, Germany), and washed once with PBS. Following this, nuclear staining was performed with 200 µL of Hoechst 33342 reagent (Invitrogen, Eugene, OR, USA) at 2 µg/mL diluted in PBS and for 10 min at room temperature. Subsequently, the plastic media chamber was detached from the slide, the gasket was removed, 10 µL of Vectashield mounting medium (Vector Laboratories, Burlingame, CA, USA) was added to each field, and a coverslip was applied. Samples were scanned manually in an Eclipse E400 fluorescence microscope (Nikon, Tokyo, Japan), equipped with an automated XY stage controlled with home-built software, a 10x objective, a DAPI filter (Ex 377/50; Em 409/LP), a FITC filter (Ex 482/18; Em 520/28), an APC filter (Ex 640/30; Em 520/28), and a monochromatic camera. The exposure times were 10 ms for the detection of Hoechst, 200 ms for MUC-1-AF488, and 2000 ms for EpCAM-AF647. The images were analyzed using ICY software (http://icy.bioimageanalysis.org/) and the enumeration was done manually.

To determine the number of epitopes detected by two anti-MUC-1 clones (EMA201 and GP1.4), and two anti-EpCAM clones (VU1D9 and Ber-EP4) by flow cytometry, were used the BD Quantibrite Beads (BD Biosciences, San José, CA, USA). Measurements were taken according to the manufacturer’s protocol. Briefly, 106 cells were resuspended in 750 µL of PBS containing 20% AB-Serum (Bio-Rad, Germany) and incubated for 20 min at 37 °C (to block unspecific Ab binding), centrifuged, and resuspended in 100 µL of PBS, containing 10% AB-serum and one of the following unconjugated primary mouse anti-human antibodies: Anti-MUC-1 clone EMA 201 at 2 µg/mL (Abnova, Taipei, Taiwan, China); Anti-MUC-1 clone GP 1.4 at 2 µg/mL (Invitrogen, Carlsbad, CA, USA); Anti-EpCAM clone VU1D9 at 2.2 µg/mL (Kindly provided by Prof. Leon Terstappen); and Anti-EpCAM clone Ber-EP4 at 1.9 µg/mL (Dako, Glostrup, Denmark). Staining was performed for 30 min at 37 °C. After washing, cells were resuspended in 100 µL of PBS with 10% AB-serum containing Phycoerythrin (PE)-conjugated Rat anti-mouse lgk light chain secondary antibody clone 187.1 at 0.01 mg/mL (BD Pharmingen, San Diego, CA, USA), and incubated for 30 min at 37 °C. After washing, cells were resuspended in PBS and PE intensity was analyzed by flow cytometry on a FACSCanto (BD Biosciences, San José, CA, USA).

### 4.3. Enrichment of Cells Using the IsoFlux System

The processing of samples in the IsoFlux system (Fluxion Biosciences, CA, USA) was performed according to the standard manufacturer protocol and using the low volume holder to recover samples. Three different types of beads/kits commercially available from Fluxion Biosciences were tested for enrichment in the system (Table 1); the amount and coupling of the different beads was conducted according to the respective protocols. In the absence of staining, following enrichment, the output sample from the low volume recovery holder was resuspended in 100 µL of IsoFlux binding buffer and then transferred directly unto one field of a 14 mm 3-field adhesive slide for microscopy (Erie Scientific LLC, Portsmouth, NH, USA). The holder was subsequently washed twice with 100µL of binding buffer (final volume in the slide field was 300 µL). Subsequently, 20 µL of Vectashield mounting medium (Vector Laboratories, Burlingame, CA, USA) was pipetted over the samples and the water content of the sample was allowed to evaporate overnight at room temperature, protected from the light. In case of staining, enriched cells were similarly treated, but recovered into a 1.5 mL tube and processed manually according to the protocol of the IsoFlux Circulating Tumor Cell Enumeration Kit (Fluxion). Subsequently, cells were also transferred unto a microscope slide and treated as described above. On the next day, a coverslip was applied and the samples were scanned automatically in an Eclipse E400 fluorescence microscope (Nikon, Japan), equipped with an automated XY stage controlled with home-built software, a 10x objective, a DAPI filter (Ex 377/50; Em 409/LP), a FITC filter (Ex 482/18; Em 520/28), an APC filter (Ex 640/30; Em 520/28), and a monochromatic camera. Exposure times were 10 ms for the detection of Hoechst, 200 ms for MUC-1-AF488, and 2000 ms for EpCAM-AF647. Images were analyzed using ICY software (http://icy.bioimageanalysis.org/) and enumeration was done manually.

### 4.4. Enrichment of Cells Using the KingFisher Duo Prime Purification System

Different programs were designed to enrich rare cells from the mimicked-DLA product (see Figure S3) and these were used in different experiments in the present work, as indicated. Four different types of magnetic beads commercially available from Thermo Fisher Scientific were tested for enrichment in the system (Table 1).

For each bead type, we have tested three different amounts of beads (MIN, MID, and MAX) (see Table 1, Table S1, Figures S1 and S2). Coupling of the beads was conducted according to the respective manufacturer protocol, and the resulting coupled beads were resuspended in 200 µL of binding buffer (0.1% BSA, 2 mM EDTA in PBS). Beads, sample and buffers for the enrichment protocol were added to a Microtiter DeepWell 96 plate (Thermo Fisher Scientific, Dreieich, Germany) and the enrichment was executed according to the protocol scheme (See Figure S3). For the “WuDuo1S” and “WuDuo2S” protocols, the antibody mix used for staining samples was composed of AF647-conjugated anti-CD45 clone HI30 at 4 µg/mL (Biolegend, San Diego, CA, USA), AF488-conjugated anti-CK19 clone A53-B/A2 at 3.5 µg/mL (Exbio, Czech Republic), AF488-conjugated anti-panCK clone C11 at 3.5 µg/mL (Abcam, Cambridge, United Kingdom), in 1x BD Perm/wash (BD Biosciences, San Diego, CA, USA), in a total volume of 200 µL. In these two protocols, the nuclear staining solution was Hoechst 33342 (Invitrogen, Eugene, OR, USA) at 2 µg/mL, diluted in PBS. After enrichment/staining, the sample (130 µL) was transferred unto one field of a 14 mm 3-field adhesive slide for microscopy (Erie Scientific LLC, Portsmouth, NH, USA), and the sample well was further washed twice with 85 µL (the total volume in the slide field was 300 µL). The sample on the slide was treated and scanned automatically, as described above.

## 5. Conclusions

In conclusion, here, we demonstrate that both IsoFlux and KingFisher systems can enrich rare cells spiked in high concentrated blood samples, but the KingFisher system offers a set of user-definable features that, combined, are unique in the CTC field: the possibility of using different beads, different epitopes, automated protocols for sequential steps of enrichment, automated protocols combining enrichment and staining, and automated protocols combining depletion and positive enrichment further expand the applicability of the instrument. Furthermore, the good performance, the low costs and the high throughput makes the system suitable for the systematic enrichment of CTCs from clinical DLA samples (Table 2).

## Figures and Tables

**Table 1:** Beads used for enrichment on the IsoFlux and KingFisher systems.

Type of Beads		IsoFlux			Thermo Fisher			
		Iso-CEK	Iso-RCEK	Iso-RCIK-SA	Dy-EpE	Dy-ACK	Dy-BioB	Pi-Strep
Commercial name		CTC Enrichment Kit	Rare Cell Enrichment Kit	Rare Cell Isolation Kit SA	Dynabeads Epthelial Enrich	Dynabeads Antibody Coupling Kit	Dynabeads Biotin Binder	Pierce Streptavidin Beads
Diameter (µm)		4.2 *	4.2 *	3.0 *	4.5	2.8	2.8	1
Concentration (beads/mL)		0.27 **	n.a.	n.a.	4 × 10^8^	6.7 × 10^8^	4 × 10^8^	96 × 10^8^
Coupling		Pre-coupled with anti-EpCAM Ab	For coupling with mouse IgG antibodies	For coupling with Biotin-conjugated Abs	Pre-coupled with anti-EpCAM Abs	For coupling via covalent binding	For coupling with Biotin-conjugated Abs	For coupling with Biotin-conjugated Abs
Coupled clone	EpCAM	n.a.	BerEP4 VU1D9	VU1D9	BerEP4	VU1D9	VU1D9	VU1D9
	MUC-1	-	EMA201 GP1.4	Not tested	-	Not tested	GP1.4	Not tested
Amount of beads	According to protocol	40µL	50µL	62.5µL	-	-	-	-
	MIN	-	-	-	2.4 µL	3.7 µL	6.2 µL	2 µL
	MID	-	-	-	12 µL	18.5 µL	31 µL	10 µL
	MAX	-	-	-	24 µL	37 µL	62 µL	19.9 µL

n.a.—Information not available. *— Information determined experimentally (Please see Figure S1). **- Information determined experimentally (Please see Figure S2). Amount of beads refers to the volume of the commercially available bead suspensions as provided by the manufacturer which was used per sample.

**Table 2:** Resume of advantages and disadvantages found for both systems for CTC enumeration.

**Isoflux**	
**Advantages**	**Disadvantages**
Easy to use Some flexibility concerning the type of beads	One single running modus Staining is done manually
**KingFisher**	
**Advantages**	**Disadvantages**
Easy to use Inexpensive technology The running protocol can be customized and it can include multiple steps for enrichment with different epitopes, depletion of CD45 cells, and subsequent staining of samples Up to 96 samples can be run in parallel Flexibility concerning the type of beads Beads available combining high recovery and possibility of staining	Reagents are not provided as a kit Any change in the running protocol requires validation

## Supplementary Materials

The following are available online at https://www.mdpi.com/2072-6694/12/4/933/s1: Table S1: Different amounts of Thermo Fisher beads used for enrichment in the KingFisher system, Figure S1: Estimation of the size of Iso-CEK and Iso-RCEK beads, Figure S2: Determination of the concentration of the Iso-CEK beads, Figure S3: Plate setup and protocols used for enrichment of CTCs in the KingFisher instrument, Figure S4: Recovery of CAPAN-1 and HuP-T4 using the CellSearch system, Figure S5: Recovery of HuP-T4 and CAPAN-1 with four different types of beads, Figure S6: Effect of cell preservative in the recovery of CAPAN-1 cells, Figure S7: Recovery of CAPAN-1 cells spiked in whole blood samples, and recovery of colon and breast cancer cell lines spiked in mimicked-DLA products, Figure S8: Determination of the number of white blood cells co-enriched in KingFisher system using BioBMAX-VU1D9 beads, Figure S9: EpCAM-based enrichment after depletion of CD45pos cells, Figure S10: Capturing of staining antibodies by the beads.
