# Supplementary material for: Magnetic-Based Enrichment of Rare Cells from High Concentrated Blood Samples
Source: Cancers (Basel). 2020 Apr 10;12(4):933. doi: 10.3390/cancers12040933 (PMC7225976; doi:10.3390/cancers12040933)
Supplement: Supplementary file 1 [file cancers-12-00933-s001.pdf]

Supplementary Materials:

Magnetic-Based Enrichment of Rare Cells from High Concentrated Blood Samples

Junhao Wu, Katharina Raba, Rosa Guglielmi, Bianca Behrens, Guus Van Dalum, Georg Flügen, Andreas Koch, Suraj Patel, Wolfram T. Knoefel, Nikolas H. Stoecklein and Rui P. L. Neves

Table 1. Different amounts of Thermo Fisher beads used for enrichment in the KingFisher system.

		**Type of Beads**							
		**Dy-EpE**		**Dy-ACK**		**Dy-BioB**		**Pi-Biot**	
**Amount**	**Total surface area**	Number of beads	Volume from original bead suspension	Number of beads	Volume from original bead suspension	Number of beads	Volume from original bead suspension	Number of beads	Volume from original bead suspension
Minimal (MIN)	6.1×10^7 μm^2	9.6×10^5	2.4 μL	24.8×10^5	3.7 μL	24.8×10^5	6.2 μL	191.5×10^5	2.0 μL
Middle (MID)	5× MIN = 30.6×10^7 μm^2	19.2×10^5	12.0 μL	124×10^5	18.5 μL	124×10^5	31.0 μL	957.7×10^5	10.0 μL
Maximal (MAX)	10 × MIN = 61.1×10^7 μm^2	38.4×10^5	24.0μL	248×10^5	37.0 μL	248×10^5	62.0 μL	1915.4×10^5	19.9 μL

Notes: The minimal (MIN) surface area is equivalent to the total surface area provided by the Iso-CEK beads in a standard IsoFlux CTC Enrichment Kit assay, which was calculated upon determination of the size (Figure S2) and number (Figure S3) of Iso-CEK beads used in an IsoFlux CTC Enrichment Kit assay. Middle surface area (MID) was subsequently defined as 5xMIN, and the maximal surface area (MAX) was defined as 10xMIN.

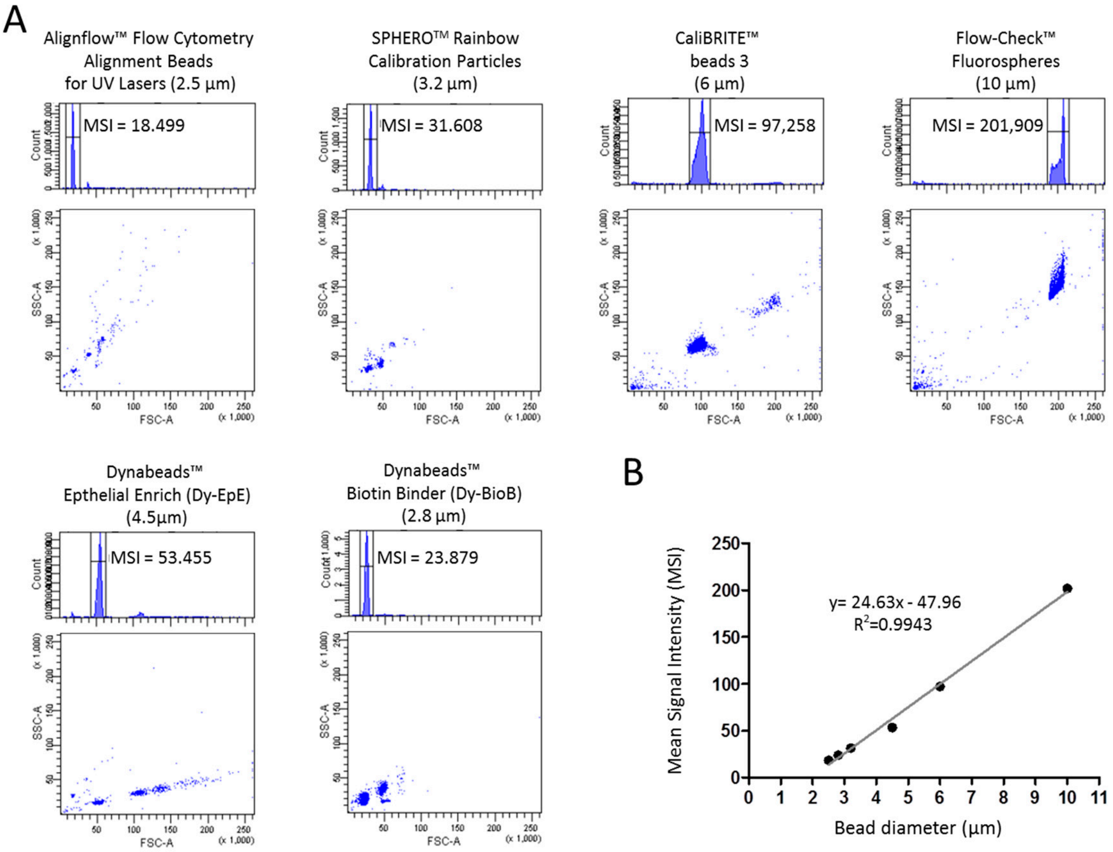

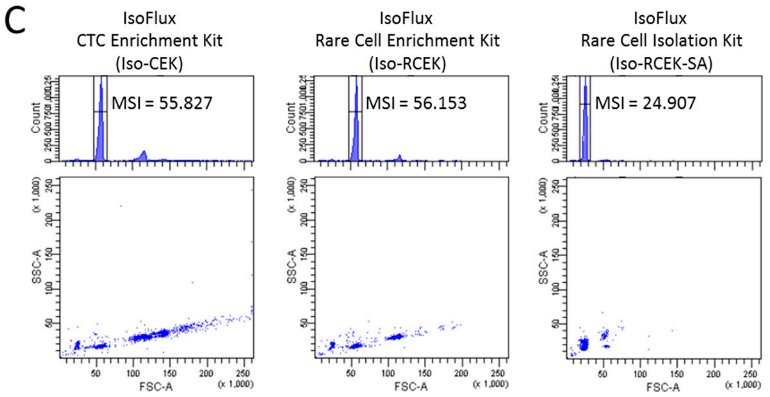

D

Beads	Measured FSC MSI	Calculated mean diameter
Iso-CEK	55.827	4.21
Iso-RCEK	56.153	4.23
Iso-RCEK-SA	24.907	2,96

Figure S1. Estimation of the size of Iso-CEK, Iso-RCEK and Iso-RECK-SA beads. **(A)** Forward (FSC) and Side Scatter (SSC) dot plots and Forward scatter (FCS) histograms obtained by flow cytometry in a FACSCanto (BD) (SSC at a voltage of 346 V and FSC at 220 V) for six beads with known sizes: (i) Alignflow Flow Cytometry Alignment Beads for UV Lasers (Molecular Probes, Oregon, USA, #A16502) with 2.5 µm; (ii) SPHERO Rainbow Calibration Particles (Spherotech, #RCP-30-5A-2) with 3.2 µm; (iii) CaliBRITE beads 3 (BD, #340486) with 6 µm; (iv) Flow-Check Fluorospheres (Beckman Coulter, CA, USA, #6605359), with 10 µm; (v) Dy-EpE with 4.5 µm; (vi) Dy-BioB with 2.8 µm. For each type of bead the mean signal intensity (MSI) of the single bead population depicted in the histograms was determined. **(B)** Linear regression calculated from the correlation between diameter and FSC-MSI of the beads in (A). **(C)** Flow cytometry analysis of the Iso-CEK, Iso-RCEK, and Iso-RCEK-SA beads as done in (A). **(D)** Calculation of approximate bead diameter using the equation of the linear regression established in (B).

A

Dilution from original Dy-EpE bead suspension ($4x10^8$/ml)	Dy-EpE bead concentration ($x10^8$/ml)	Measured OD600
1:15	0.266667	28.415
1:16	0.250000	28.676
1:17	0.235294	28.207
1:20	0.200000	21.057
1:25	0.160000	19.560
1:30	0.133333	13.999
1:35	0.114286	12.442
1:40	0.100000	11.495
1:45	0.088889	10.174

B

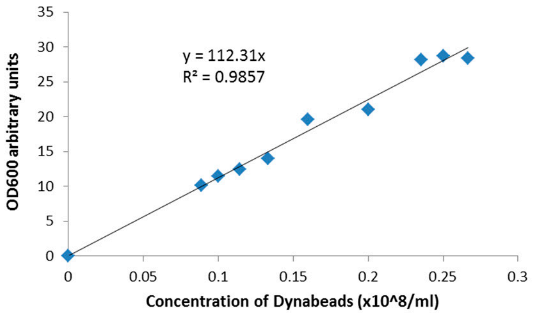

C

Dilution from Iso-CEK bead suspension	Measured OD600	Calculated Iso-CEK bead concentration in the original suspension ((OD600/112.31)*dilution)
1:1	27.404	$0.244 x10^8$/ml
1:2	16,060	$0.286 x10^8$/ml
1:4	8,171	$0.291 x10^8$/ml
	Mean:	$0.274 x10^8$/ml

D

Calculated concentration of Iso-CEK beads in the original bead suspension (C)	$0.274 x10^8$/ml
Volume of bead suspension used per sample in the IsoFlux CTC Enrichment Kit according to protocol (V)	40 µl
Calculated number of beads used per sample in the IsoFlux CTC Enrichment Kit assay (N=C*V)	$10.96 x10^5$ beads
Calculated concentration of beads used per sample (N/V)	$10.96 x10^5$/ml
Calculated diameter of Iso-CEK beads (from SupplementalFigure 2)	4,2143 µm
Surface area of a single bead ($A = 4\pi r^2$)	55.79 μm^2
Total surface area available for interaction with cells in IsoFlux CTC Enrichment Kit assay (Total Area=N*A)	$6.11 x10^7$ μm^2

Figure S2. Determination of the concentration of the Iso-CEK beads and the surface area of Iso-CEK beads available for interaction with cells in the IsoFlux CTC Enrichment Kit assay. The concentration of Iso-CEK beads was determined by comparing the optical density at 600 nm (OD600) of Iso-CEK bead suspensions with that of Dynabeads Epithelial Enrich (Thermo Fisher Scientific, #16102) bead suspensions with similar nominal size. (**A**) OD600 of a series of dilutions from the original Dy-EpE bead suspension at $4x10^8$ beads/ml. OD600 was measured in a BioPhotometer (Eppendorf, Germany) using disposable cuvettes (Sarstedt, Germany). (**B**) Standard curve made with the measurements in (A). (C) OD600 of a three dilutions from the original Iso-CEK bead suspension and the concentration of Iso-CEK beads in the original suspension calculated based on the formula of the standard curve obtained in (**B**). OD600 was measured as in (**A**). (**D**) Parameters and steps used to calculate the total surface area of the beads that is available for interaction with cells in a standard IsoFlux CTC Enrichment Kit assay where Iso-CEK beads are employed.

Plate	Row	WuDuo protocol 1	WuDuo protocol 1S	WuDuo protocol 2	WuDuo protocol 2S	Well Content	Step Description	Instructions
Plate A	A	(1)	(1)	(1)	(1)	Tips	Picking the tips for magnet protection	
	B	(2)	(2)	(2)	(2)	Suspension of beads A (200 µl)	Collection of beads	5times, every time 30s
	C	(3)	(3)	(3)	(3)	Binding buffer (200 µl)	Washing of beads	30s pause then collect as in row B
	D	(4)	(4)	(4) (9)	(4) (11)	Sample (1000 µl)	Enrichment of CTCs	36s speed slow then 36s pause with 100 loops
	E	(5)	--	(5)	--	Binding buffer (200 µl)	Washing of enriched cells	30s pause then collect as in row B
	F	--	(5)	--	(5)	Antibody mix (200 µl)	Immunostaining of enriched cells	45min pause then collect as in row B
	G	--	(6)	--	(6)	Hoechst solution (200 µl)	Nuclear staining of enriched cells	2min pause then collect as in row B
	H	--	--	--	--			
	Stripe	(6)	(7)	(6)	(7)	Binding buffer (130 µl)	Recovery of sample	3min speed slow
Plate B	A	--		(7)	(8)	Tips	Picking the tips for magnet protection	
	B	--		(8)	(9)	Suspension of beads B (200 µl)	Collection of beads	5times, every time 30s
	C	--		(10)	(10)	Binding buffer (200 µl)	Washing of beads	30s pause then collect as in row B
	D	--		(11)	--	Binding buffer (200 µl)	Washing of enriched cells	30s pause then collect as in row B
	E	--		--	(12)	Antibody mix (200 µl)	Immunostaining of enriched cells	45min pause then collect as in row B
	F	--		--	(13)	Hoechst solution (200 µl)	Nuclear staining of enriched cells	2min pause then collect as in row B
	G	--		--	--			
	H	--		--	--			
	Stripe	--		(12)	(14)	Binding buffer (130 µl)	Recovery of sample	3min speed slow

Figure S3. Plate setup and protocols used for enrichment of CTCs in the KingFisher instrument. Illustration of the 4 WuDuo protocols (WuDuo 1, 1S, 2 and 2S) including the content of the plate, description of each step and the KingFisher settings. The sequence of the steps in the respective protocol (each row) is indicated by the number (n) next to the illustration of each well. The cups items used in this figure were extracted from "Medical Equipment" attributed to Servier Medical Art. These were used and adapted under a Creative Commons Attribution 3.0 Unported License.

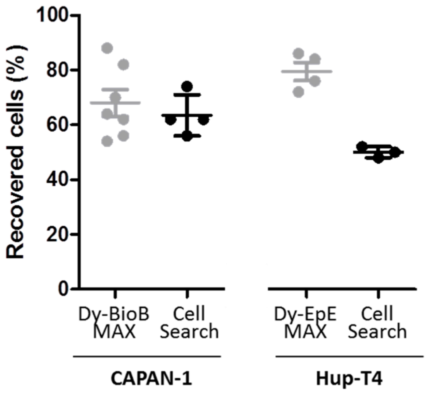

Figure S4. Recovery of CAPAN-1 and HuP-T4 using the CellSearch system. One day prior to the experiment, $1x10^8$ PBMNCs were resuspended in 8 mL of PBS containing 0.1% BSA and 2 mM EDTA, transferred into a CellSave preservative tube (Menarini Silicon Biosystems, Huntingdon Valley, PA, USA), and incubated overnight at room temperature on a rotator. Separately, CAPAN-1 or HuP-T4 cells harvested from culture were treated similarly. On the next day, 50 cell line cells were spiked by flow cytometry into the CellSave tube containing the PBMNCs, enriched with the CellSearch CTC Kit in the CellTrack Autoprep (Menarini Silicon Biosystems, San Diego, CA, USA), and enumerated in the CellTrack Analyzer II (Menarini Silicon Biosystems) according to the manufacturer standard procedure. Recovery data from cells with Dy-BioB and Dy-EpE beads (in gray in the graph) is the same as in Figure 1 of the main manuscript.

A

Cells recovered	Hup-T4 1	Hup-T4 10	Hup-T4 30	Hup-T4 50	Hup-T4 100	CAPAN-1 1	CAPAN-1 10	CAPAN-1 30	CAPAN-1 50	CAPAN-1 100
**Iso-CEK**	1	3	13	19	56	0	5	15	30	37
	1	6	19	18	51	1	4	14	23	43
	1	4	21	28	62	1	4	6	20	42
				35					17	
				25					25	
				36						
**Iso-RCEK (BerEP4)**	1	5	18	31	53	1	6	9	25	26
	1	2	15	22	49	1	3	13	14	31
	1	3	14	19	36	1	2	11	21	43
				23					19	
				31					21	
**Dy-EpE^{MID}**	1	6	18	37	80	0	3	17	31	52
	1	4	22	37	78	0	5	12	38	47
	1	9	17	41	74	1	5	13	23	48
				35					28	
**Dy-BioB^{MAX} (VU1D9)**		6	14	34	52		2	11	27	49
		6	24	31	45		4	18	44	50
		8	19	30	42		5	12	35	49
				36					41	
									32	
									28	
									31	

B

Hup-T4

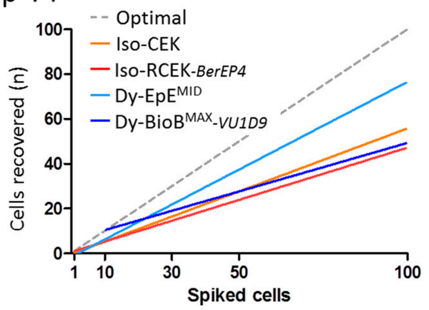

	R^2	Slope	Recovery
**Optimal**	1.000	1.000 ± 0.0	
**Iso-CEK**	0.9360	0.5615 ± 0.03672	56 %
**Iso-RCEK**	0.9276	0.4659 ± 0.03361	47 %
**Dy-EpE**	0.9913	0.7817 ± 0.01956	78 %
**Dy-BioB**	0.8938	0.4326 ± 0.04497	43 %

Capan-1

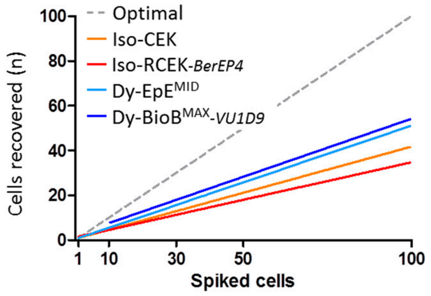

	R^2	Slope	Recovery
**Optimal**	1.000	1.000 ± 0.0	
**Iso-CEK**	0.9427	0.4120 ± 0.02622	42 %
**Iso-RCEK**	0.8896	0.3353 ± 0.03051	34 %
**Dy-EpE**	0.9513	0.5076 ± 0.03070	51 %
**Dy-BioB**	0.8411	0.5178 ± 0.06016	52 %

Figure S5. Recovery of HuP-T4 and CAPAN-1 with four different types of beads. One, ten, 30, 50, and 100 HuP-T4 or CAPAN-1 cells pre-labelled with CellTracker Green CMFDA Dye (Thermo Fisher Scientific) were spiked in pseudo DLA products. Enrichment was done with Iso-CEK and Iso-RCEK-*BerEP4* in the IsoFlux system and with Dy-EpE and Dy-BioB^{MAX}-*VU1D9* in the KingFisher system. **(A)** Number of cells recovered in each experimental replicate. **(B)** Linear regression of the data listed in (A) with information on the linearity (R^2) and slope. Recovery along the range of experiments was determined from the slope (Recovery = Slope × 100).

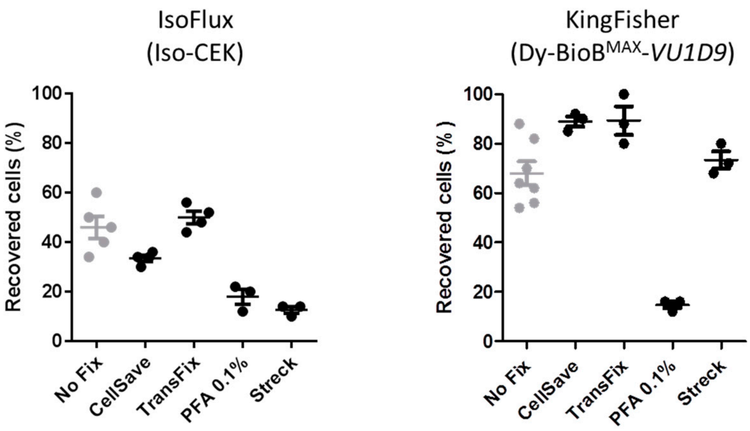

Figure S6. Effect of cell preservative in the recovery of CAPAN-1 cells. 1× 10^8 PBMNCs resuspended in 8 mL of PBS containing 0.1% BSA and 2 mM EDTA were transferred into CellSave preservative tube (Menarini Silicon Biosystems, Huntingdon Valley, PA, USA), TransFix Vacuum tube (ref. TVT-09-50-45, Cytomark, Buckingham, UK), Cell-Free DNA BCT CE tube (Streck, La Vista, NE, USA) or a 15 mL conical centrifuge tube (Greiner bio-one, DE) containing 500 µL of 1.6% Paraformaldehyde (PFA) (final PFA concentration 0.1%). Tubes were incubated overnight at room temperature on a rotator. Separately, cell line cells were resuspended in 8 mL of PBS containing 0.1% BSA and 2 mM EDTA and treated similarly. On the next day, the PBMNCs samples were pelleted upon centrifugation at 400 g for 7 min, the volume of the sample was adjusted to 850 µL with PBS containing 0.1% BSA and 2 mM EDTA, and cell line cells were spiked by flow cytometry. Spiked samples were then analyzed in the IsoFlux system using the Iso-CEK beads or KingFisher system using the Dy-BioB-*VU1D9* beads. Recovery data from cells without fixation (in gray in the graph) is the same as in Figure 1 of the main manuscript.

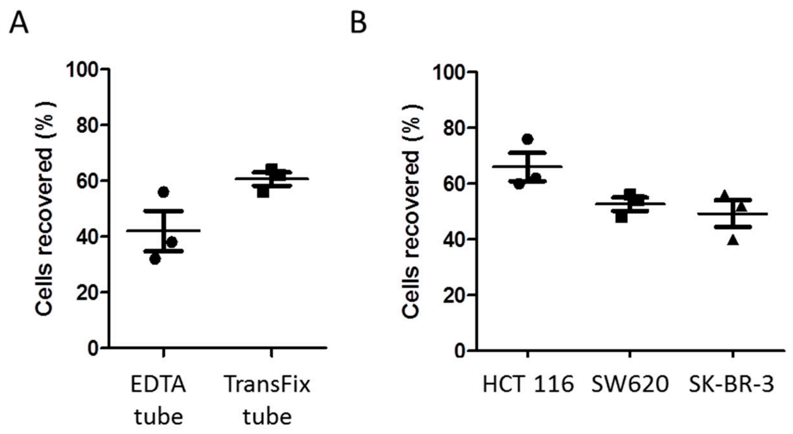

Figure S7. Recovery of CAPAN-1 cells spiked in whole blood samples, and recovery of colon and breast cancer cell lines spiked in pseudo DLA products. (A) Recovery of CAPAN-1 cells spiked in whole blood samples. 7.5 mL peripheral blood from healthy donors and collected in EDTA or TransFix tubes was treated with Red Blood Cell Lysis buffer (G-Biosciences, MO, USA) (22.5 mL and 30 mL, respectively) for 10 min at room temperature in rotation. Subsequently samples were centrifuged at 500 g for 10 min at room temperature, the supernatant was discarded, and the pellet was resuspended in 850 µL of binding buffer (0.1% BSA, 2 mM EDTA in PBS) and finally transferred into a Microtiter DeepWell 96 plate (Thermo Fisher Scientific, Germany). 50 CAPAN-1 cells pre-labelled with CellTracker Green CMFDA Dye were spiked by flow cytometry using the MoFlo XDP flow cytometer (Beckman Coulter, Krefeld, Germany) into the samples. Enrichment in the KingFisher system was executed according the WuDuo1 protocol. (B) Recovery of colon and breast cancer cell lines spiked in pseudo DLA products. 50 HCT 116, SW620 and SK-BR-3 cells pre-labelled with CellTracker Green CMFDA Dye were spiked by flow cytometry into pseudo DLA products prepared as described in the main manuscript.

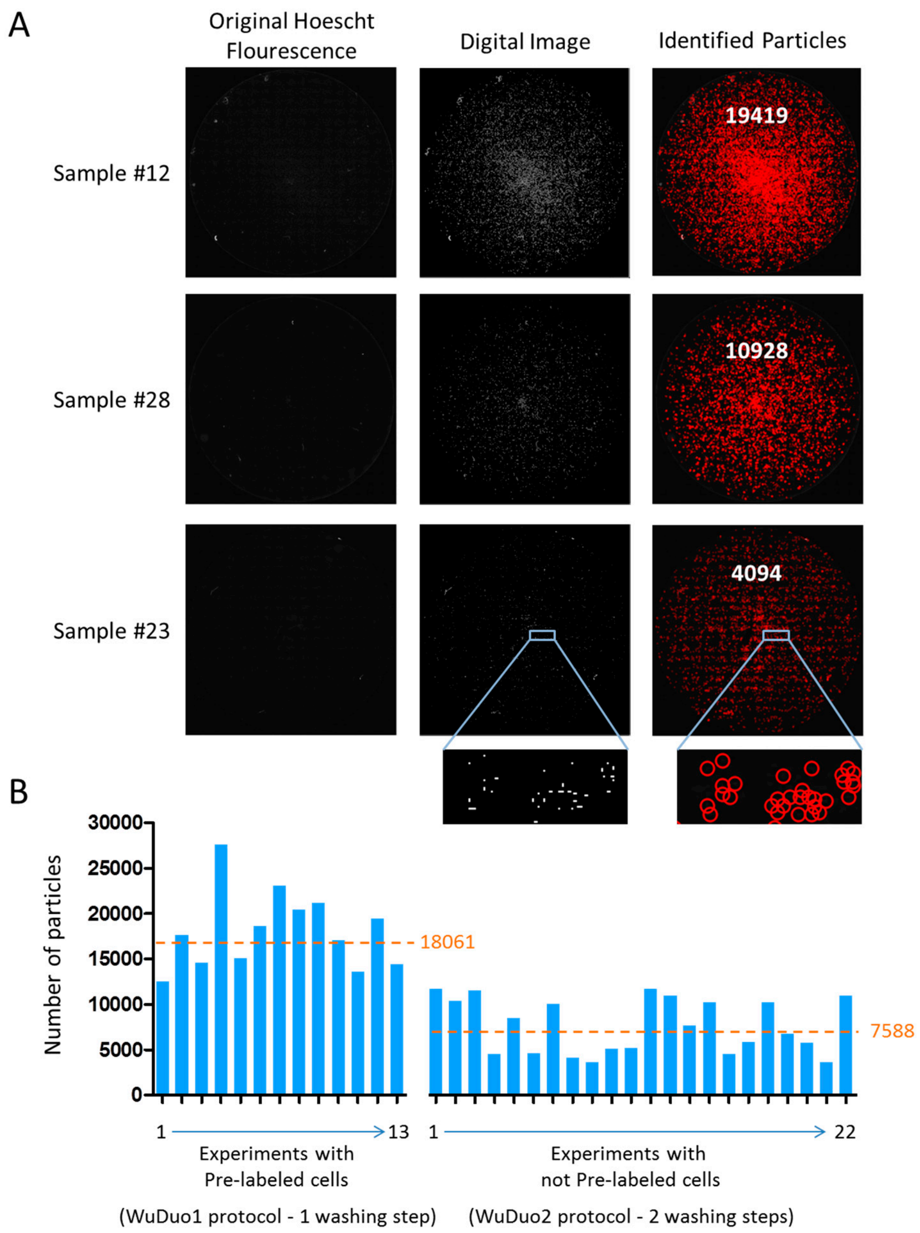

Figure 8. Determination of the number of white blood cells co-enriched in KingFisher system using BioB^{MAX}-VU1D9 beads. To determine the total number of WBCs carried, we marked all enriched cells with Hoechst nuclear dye, making possible their subsequent identification and enumeration by fluorescence microscopy. For the staining we added Hoechst 33342 (Invitrogen, OR, USA) at 2 µg/mL to the binding buffer in well E of the WuDuo1 protocol or well G of WuDuo1S protocol. After enrichment, the sample was processed as described in the main section of the manuscript and the enriched samples were analysed by fluorescence microscopy. The images obtained were subsequently analysed with ICY software (http://www.icy.bioimageanalysis.org/) and the number of Hoechst positive cells was determined using spot detector plugin of ICY. **(A)** Overview montage of all images obtained from Hoescht flourescence in three representative samples. The different panels show the original fluorescence image (left), the digital image after processing with ICY software (middle), and then particles detected by the spot detector plugin (right). **(B)** Number of particles detected in experiments performed with the WuDuo1 and WuDuo1S protocols. The mean values are indicated and represented by the dashed lines.

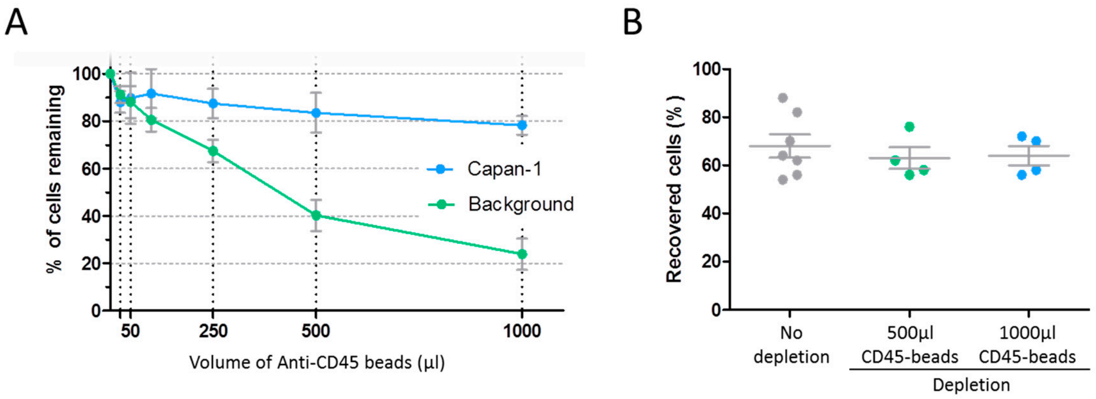

Figure S9. EpCAM-based enrichment after depletion of $CD45^{pos}$ cells. **(A)** 1×10^5 CAPAN-1 cells pre-labeled with CellTracker Green CMFDA Dye in suspension were spiked into pseudo DLA products (1 mL at 1×10^8 MNCs/mL). CD45 Depletion was performed in the KingFisher system using different volumes of Dynabeads CD45 magnetic bead suspension (Life Technologies by Thermo Fisher Scientific, Vilnius, Lithuania). After depletion, the volume of the sample was adjusted to 1 mL with binding buffer, mixed with 50 µL of CountBright absolute counting beads (0.52×10^5 beads/50 µL) (Invitrogen by Thermo Fisher Scientific, Eugene, OR, USA) and analyzed by flow cytometry in a FACSCanto (BD Biosciences, San José, , CA, USA). Acquisition was stopped when 2600 or 5200 beads (5% or 10% of the sample) were detected. The number of CellTracker Green positive events (spiked cells) and CellTracker Green negative events (WBCs) was determined. In order to calculate the faction depletion (percentage as plotted), the number of cells of the two populations was correlated with the respective populations in samples where no CD45 beads were used (control). **(B)** Recovery of CAPAN-1 cells pre-labeled with CellTracker Green CMFDA Dye after CD45 depletion and subsequent EpCAM enrichment. 500 µL and 1000 µL Dynabeads CD45 magnetic beads were used for depletion. In gray, for comparison, is the same data as in Figure 1 of the main manuscript obtained without depletion.

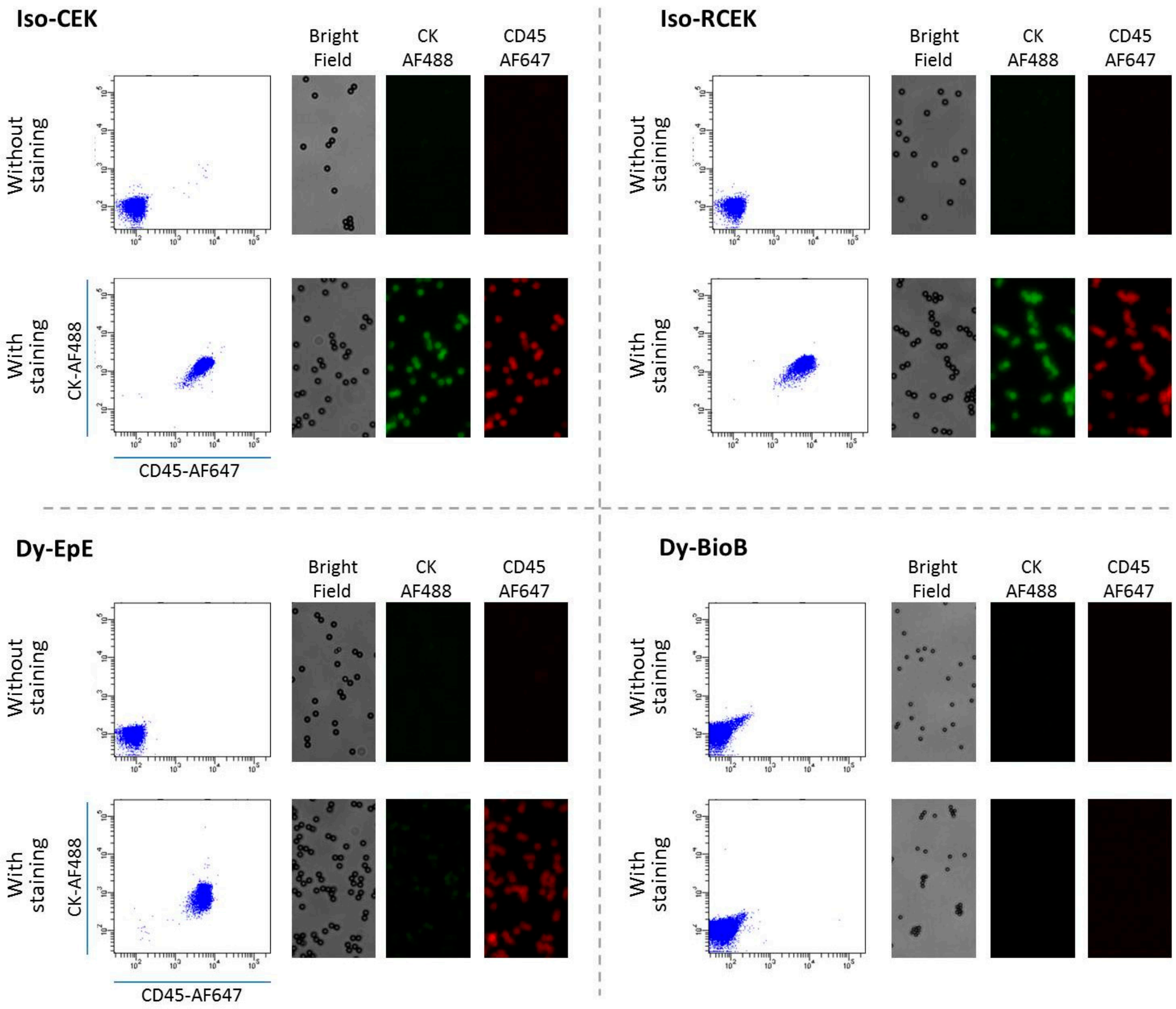

Figure S10. Capturing of staining antibodies by the beads. Flow cytometry and fluorescence microscopy analyses of beads with and without incubation with the complete staining cocktail for clinical samples. Staining was done incubating the beads 40 min in 200 μL of the staining solution and subsequently washing beads once in PBS containing 0.5% BSA and 2 mM EDTA. Flow cytometry analyses were done using a FACSCanto (BD Biosciences, San José, CA, USA). CK-AF488 was analyzed in the FITC channel (Ex 488; Em 530/30) with a voltage of 401 V, and CD45-AF647 in the APC channel (Ex 633; Em 660/20) with a voltage of 456 V. Microscopy analyses were done using an Eclipse E400 (Nikon, Tokyo, Japan) inverted microscope equipped with a 20x objective and a FITC filter (Ex 482/18; Em 520/28) and an APC filter (Ex 640/30; Em 520/28). The exposure time for bright field imaging was 25 ms and for fluorescent imaging 400 ms for both filters.
